# Geographical and temporal distribution of multidrug-resistant *Salmonella* Infantis in Europe and the Americas

**DOI:** 10.3389/fmicb.2023.1244533

**Published:** 2024-02-13

**Authors:** Jaromir Guzinski, Joshua Potter, Yue Tang, Rob Davies, Christopher Teale, Liljana Petrovska

**Affiliations:** Animal and Plant Health Agency, Addlestone, Surrey, United Kingdom

**Keywords:** *Salmonella* Infantis, antimicrobial resistance genes, pESI-like megaplasmid, whole-genome sequencing, phylogenomics, population genetic structure

## Abstract

Recently emerged *S.* Infantis strains carrying resistance to several commonly used antimicrobials have been reported from different parts of the globe, causing human cases of salmonellosis and with occurrence reported predominantly in broiler chickens. Here, we performed phylogenetic and genetic clustering analyses to describe the population structure of 417 *S.* Infantis originating from multiple European countries and the Americas collected between 1985 and 2019. Of these, 171 were collected from 56 distinct premises located in England and Wales (E/W) between 2009 and 2019, including isolates linked to incursions of multidrug-resistant (MDR) strains from Europe associated with imported poultry meat. The analysis facilitated the comparison of isolates from different E/W sources with isolates originating from other countries. There was a high degree of congruency between the outputs of different types of population structure analyses revealing that the E/W and central European (Germany, Hungary, and Poland) isolates formed several disparate groups, which were distinct from the cluster relating to the United States (USA) and Ecuador/Peru, but that isolates from Brazil were closely related to the E/W and the central European isolates. Nearly half of the analysed strains/genomes (194/417) harboured the IncFIB(pN55391) replicon typical of the “parasitic” pESI-like megaplasmid found in diverse strains of *S.* Infantis. The isolates that contained the IncFIB(pN55391) replicon clustered together, despite originating from different parts of the globe. This outcome was corroborated by the time-measured phylogeny, which indicated that the initial acquisition of IncFIB(pN55391) likely occurred in Europe in the late 1980s, with a single introduction of IncFIB(pN55391)-carrying *S.* Infantis to the Americas several years later. Most of the antimicrobial resistance (AMR) genes were identified in isolates that harboured one or more different plasmids, but based on the short-read assemblies, only a minority of the resistance genes found in these isolates were identified as being associated with the detected plasmids, whereas the hybrid assemblies comprising the short and long reads demonstrated that the majority of the identified AMR genes were associated with IncFIB(pN55391) and other detected plasmid replicon types. This finding underlies the importance of applying appropriate methodologies to investigate associations of AMR genes with bacterial plasmids.

## 1 Introduction

*Salmonella enterica* subsp. *enterica* serovar Infantis (*S.* Infantis) is one of over 2,600 recognised *S. enterica* serovars (Issenhuth-Jeanjean et al., 2014). This serovar is non-host specific but has been widely associated with the poultry production chain in several European countries and globally and has the propensity for horizontal transmission of mobile genetic elements (MGEs) (EFSA Panel on Biological Hazards et al., 2019). In the period of 2018–2020, *S.* Infantis was the most reported serovar from broilers within the EU including from both animal and meat samples (European Food Safety Authority [EFSA] and European Centre for Disease Prevention and Control [ECDC], 2021a,b). In the United Kingdom (UK), *S.* Infantis, where it is one of six serovars that are regulated in breeding flocks of chickens under the National Control Programme, was less frequently detected in broiler chickens, laying hen flocks, and adult chicken breeding flocks than other *Salmonella* serovars in the period of 2017–2021 (APHA, 2022). *Salmonella* Infantis has also been associated with livestock such as pigs or cattle in some countries (Gymoese et al., 2019; Newton et al., 2020). For several years, *S.* Infantis has consistently been identified in the top five in Europe and the top ten in North America of all *S. enterica* serovars causing non-typhoidal salmonellosis in humans (Centers for Disease Control and Prevention [CDC], 2017; European Food Safety Authority [EFSA] and European Centre for Disease Prevention and Control [ECDC], 2021b).

*Salmonella* Infantis does not appear to be more virulent than other serovars and typically causes non-life-threatening episodes of gastroenteritis. However, certain strains may show enhanced virulence, and invasive infections can lead to serious complications such as bacteraemia or meningitis and require antimicrobial therapy (EFSA Panel on Biological Hazards et al., 2019). Ciprofloxacin is an important antimicrobial for treating salmonellosis in adults, and β-lactams (e.g., ampicillin or third-generation cephalosporins) have also been widely used, especially for the treatment of children (Medalla et al., 2016). In England and Wales (E/W), human salmonellosis associated with multidrug-resistant (MDR) *S.* Infantis has been increasing, with 3.5 times more cases detected between 2014 and 2018 than between 2000 and 2013 (Lee et al., 2021). There have also been reports of MDR *S.* Infantis isolations from both animal hosts and humans in other European countries, such as Hungary, Israel, Italy, Switzerland, the USA, and Brazil (Gal-Mor et al., 2010; Nógrády et al., 2012; Aviv et al., 2014; Franco et al., 2015; Hindermann et al., 2017; Tate et al., 2017; Monte et al., 2019; Proietti et al., 2020). *Salmonella* MDR strains have been shown to represent a significant public health concern due to difficulties in treating the disease, which may result in longer hospitalisation and increased mortality rates in patients infected with MDR *Salmonella* isolates, compared to patients infected with drug-susceptible strains (Helms et al., 2002; Martin et al., 2004; Su et al., 2004; Mølbak, 2005; Parisi et al., 2018).

The MDR patterns reported in *S.* Infantis vary depending on the strain and the antimicrobial testing panel. For example, in the UK, furazolidone susceptibility is routinely tested and such resistance in *S.* Infantis tends to be associated with European “epidemic” strains. A clonal MDR nalidixic acid–streptomycin–sulfamethoxazole–tetracycline (NaSSuT)-resistant strain widely circulating in the European poultry industry has been reported in Hungary, Poland, Austria, Germany, and Switzerland (Nógrády et al., 2012; Hindermann et al., 2017), whereas MDR *S.* Infantis isolates exhibiting a nalidixic acid–sulfamethoxazole–tetracycline (NaSuT) resistance pattern have been reported in Switzerland (Hindermann et al., 2017). However, of greater public health concern is the emergence of clusters of extended-spectrum beta-lactamase (ESBL)-producing *S.* Infantis strains that have been reported in Italy, Switzerland, and the United States (Franco et al., 2015; Hindermann et al., 2017; Tate et al., 2017). The ESBL-producing *S.* Infantis strains usually possess ESBLs belonging to the cefotaximase family, conferring resistance to third-generation cephalosporins and related compounds, and may also display reduced susceptibility to ciprofloxacin, often in combination with resistance in the NaSSuT pattern. Horizontal transfer of an *S.* Infantis endemic “pESI” plasmid (plasmid of emerging *S.* Infantis) into *Escherichia coli* strains and possibly also into Gram-positive mouse microbiota species was demonstrated experimentally in a mouse model (Aviv et al., 2016), and recently, it has been reported that commensal *E. coli* could potentially be regarded as a reservoir of resistance genes for *S.* Infantis in broiler chicks (Szmolka et al., 2021). The usage of antimicrobials in food animals for the treatment of disease provides a selective pressure for bacteria to become resistant or to acquire plasmids conveying antimicrobial resistance (Murphy et al., 2017). This is a particular problem if mobile genetic elements bestow a selective fitness advantage upon their bacterial host, resulting in the dissemination of resistance in bacteria in food animals, which can lead to the subsequent spread of the MDR bacterial strains to the human population (Bogomazova et al., 2020; Li et al., 2020).

In *S.* Infantis, the genetically homologous conjugative megaplasmids pESI (also termed “pESI-like”) that harbour virulence, fitness, and MDR genes were first described in Israel (Aviv et al., 2014) and subsequently in Italy (Franco et al., 2015; Alba et al., 2020), Switzerland (Hindermann et al., 2017), Hungary (Szmolka et al., 2018), Russia (Bogomazova et al., 2020), the United States (Tate et al., 2017), Latin America (Iriarte et al., 2017; Fuentes-Castillo et al., 2019), and Japan (Yokoyama et al., 2015). Acquisition of this type of plasmid is thought to have played an important role in the emergence of MDR *S.* Infantis in Israel by providing bacterial hosts with a significant fitness advantage (Aviv et al., 2014). pESI or pESI-like are large chimeric megaplasmids, typically around 280 to 300 kbp, that have evolved via recombination between, at least, IncI1 and IncP ancestral plasmid groups (Aviv et al., 2014; Franco et al., 2015; Hindermann et al., 2017; Tate et al., 2017). pESI or pESI-like megaplasmids often harbour genes that provide resistance to tetracyclines, sulfamethoxazole, and/or trimethoprim, encode the production of ESBL, and could contain genes that confer enhanced fitness upon their host such as increased mercury and oxidative stress tolerance (Aviv et al., 2014; Franco et al., 2015; Hindermann et al., 2017; Tate et al., 2017; Cohen et al., 2020).

The MDR-harbouring *S.* Infantis strains, and especially strains that have acquired pESI or pESI-like plasmids, are of substantial public health concern because of their tendency to establish in broiler chickens and affect the human population. Such strains have spread worldwide, although at the time of publishing, the UK has been less affected by MDR *S.* Infantis, likely due to stringent biosecurity measures in the broiler industry (European Food Safety Authority [EFSA] and European Centre for Disease Prevention and Control [ECDC], 2019; Newton et al., 2020) and intensive investigations of any incursions with the aim of preventing the establishment of infections in poultry. Increasing our knowledge of the worldwide *S.* Infantis population structure can play an important role in understanding and preventing the further spread of this foodborne pathogen. There has only been limited evidence from previous studies analysing *S.* Infantis population genetic structure of a correlation between the genetic clusters and the country of origin of the isolates (Gymoese et al., 2019; Alba et al., 2020; Nagy et al., 2020). The aims of the study were (1) to describe the population structure of a panel of 417 *S.* Infantis isolates originating from 15 countries/geographical regions using phylogenetic and genetic clustering analyses applied to whole-genome sequencing (WGS) data, (2) to investigate the genetic relatedness of 171 *S.* Infantis isolates originating from E/W, with the strongest emphasis on comparing and contrasting isolates collected from six premises of epidemiological interest, (3) to detect and characterise antimicrobial resistance (AMR) determinants and plasmids in the analysed isolates, including based on WGS data generated with long-read sequencing technology. A comparison of detected plasmids and AMR genes was presented for selected isolates that were analysed in previously published studies with potentially different analytical methods, and (4) to employ Bayesian analysis to reconstruct the time-measured phylogeny and evolution of a subset of isolates with respect to the presence and absence of the pESI or pESI-like megaplasmid.

## 2 Materials and methods

### 2.1 Sample selection and whole-genome sequencing

The selection of isolates for inclusion in the study was performed so that it covered a wide geographical area but with a focus on recent isolates from England and Wales. Additionally, sequence data from *S.* Infantis strains described in previous publications as MDR, including isolates that carried the pESI plasmid and were ESBL producers, were included in the study.

A total of 207 *S.* Infantis isolates sequenced at the Animal and Plant Health Agency (APHA) were collected between 2008 and 2019 as part of routine surveillance of livestock farms, outbreak investigations, control programmes, and research projects, and as a part of multi-country collaborative investigations. Two human isolates from Scotland (Scottish Microbiology Reference Laboratories) were included in the study. Of the 207 animal isolates, 204 were of sequence type (ST) 32, two were ST603, and one isolate was ST2283. The two Scottish human isolates were ST32. The APHA isolates were sequenced on either MiSeq or NextSeq benchtop Illumina sequencers after the paired-end libraries were prepared with the Illumina Nextera XT DNA Library Preparation Kit following the manufacturer’s instructions.

In addition, the WGS data of 228 *S.* Infantis isolates originating from Belgium, Brazil, Germany, Hungary, Italy, the Netherlands, Poland, Thailand, and the United States were downloaded from NCBI GenBank (Short Read Archive) using the fasterq-dump function of sratoolkit v2.9.6–1 (Leinonen et al., 2011) after searching the *Salmonella* EnteroBase online strain database (version from autumn 2019) for *S.* Infantis isolates. The downloaded data were analysed with an APHA in-house *in silico* serotyping pipeline to confirm the serovar. In all, 20 of the isolates downloaded from NCBI GenBank were typed by the pipeline as serovars other than *S.* Infantis and hence were excluded from further analyses.

In total, the WGS data from 413 *S.* Infantis isolates originating from 15 different countries/regions plus four isolates from an unknown country of origin sampled between 1985 and 2019 were included in the study (Table 1). The 417 *S.* Infantis sequences were collected from 16 different sources (Table 1), including 89 isolates originating from human clinical cases. The unbalanced structure of the dataset in terms of greater representation of isolates from certain countries/regions (Table 1) was dictated to a large extent by the study focus and inclusion of a large number of E/W isolates, as well as by the availability of genomic data that we could obtain from public databases for isolates from other localities with a profile of interest for the study. Importantly, for the time-measured phylogenetic analysis in Bayesian Evolutionary Analysis Sampling Trees (BEAST) (see below), the dataset was preselected such that the numbers of isolates from different countries/regions were more equivalent (Supplementary Table S1). Of the 207 APHA sequenced isolates, 171 were sourced from 56 distinct E/W premises from animal hosts or produce (Table 1). Between 10 and 40 isolates were recovered from three different premises, whereas a single isolate was recovered from 35 different premises. The inclusion of multiple isolates from the same poultry premises in E/W was performed to investigate within-farm and between-year genetic diversity as some premises had been infected for an extended period of time: 16 isolates from broiler farm A, 4 isolates from broiler farm B, 1 isolate from broiler farm C, 9 isolates from broiler processing plant D, 40 isolates from layer farm E, and 1 isolate from broiler farm F (the six premises of epidemiological interest). The 36 remaining isolates were of non-E/W origin and were sequenced at the APHA as part of collaborative research projects: 11 isolates from the British Isles (exact locality not known), 4 isolates from Hungary, 4 isolates from the Middle East, 13 isolates from the Netherlands that were part of Europe-wide ring trials, and 4 isolates from an unknown locality (Table 1). The human *S.* Infantis isolates that were obtained from patients from the United States were categorised in accordance with patient travel history (no travel history (“USA”), uncertain travel history (“probably USA”), and travel history to Ecuador/Peru (“Ecuador/Peru”)), as per the epidemiology data in Tate et al. (2017) and Brown et al. (2018) (Table 1).

**Table 1:** Isolates analysed in this study, with the number of isolates for each country of origin and each source specified.

Country of origin	Source																
	Broiler	Cattle	Dog	Duck	Environment	Feed	Food	Horse	Human	Layers	Pig	Poultry	Reptile	Seafood	Wild animal	Unknown	Total
Belgium																1	1
Brazil	8				9	3	15										35
British Isles																11	11
Ecuador/Peru									12								12
E/W	35	4	17	11		48		3		46	1		1		4	1	171
Germany		3			11	8	18		45			1	2		19	1	108
Hungary	3											1				4	8
Italy	5								2						1		8
Middle East	2											2					4
Netherlands					1				1							13	15
Poland							4		10								14
Scotland									2								2
Thailand														1			1
Probably USA									10								10
USA	4	1					1		7								13
Unknown																4	4
Total	57	8	17	11	21	59	38	3	89	46	1	4	3	1	24	35	417

### 2.2 Phylogenomics and population structure

Phylogenomic and genetic population structure analyses were performed on all collected sequences (Table 1) and for the 171 E/W isolates only. Initial analysis revealed two APHA sequenced Europe-wide ring trial isolates from the Netherlands that were of unknown origin (L01384-14 and S05257-14, both of which belonged to ST603) to be highly genetically differentiated from all other analysed isolates even though the *in silico* serotyping pipeline identified these two isolates as *S.* Infantis. L01384-14 and S05257-14 were excluded from all analyses unless otherwise stated. Hence, the phylogenetic analysis on the full dataset was performed on 415 isolates.

Multiple sequence alignment (MSA) was constructed by aligning the analysed genomes against the reference genome (a PacBio sequenced chromosome of isolate FSIS1502916 from Tate et al. (2017); RefSeq accession number: NZ_CP016408.1) with snippy v4.4.5.^1^ Identification and removal of recombination events were performed with Gubbins v2.4.1 (Croucher et al., 2015), and subsequently, polymorphic sites were extracted using SNP-sites (Page et al., 2016). The resulting core single-nucleotide polymorphism (SNP) alignment comprised 3,503 sites for the full dataset and 1,184 sites for the E/W dataset.

Maximum-likelihood (ML) phylogenetic trees were generated from a core SNP alignment (including the reference) in RAxML-NG v0.9.0 (Kozlov et al., 2019) using the generalised time-reversible nucleotide substitution model with GAMMA correction. Branch support was computed via 1,500 bootstrap replicates (Felsenstein’s bootstrap proportions), which was sufficiently high to achieve bootstrap convergence. The best-scoring ML trees were annotated with the metadata on the country of origin, host, year of isolation, farm code (selected E/W farms only), hierBAPS clustering level 1 (described below), and identified AMR genes and plasmids (described below) for each isolate in iTol (Letunic and Bork, 2019).

To further understand the levels of genetic relatedness amongst the studied isolates, snp-dists v0.6.3^2^ was used to compute a pairwise SNP distance matrix from the core SNP alignment. Additionally, SnapperDB (Dallman et al., 2018) software was utilised to assign an SNP address to each of the analysed isolates. Isolates that shared an SNP address up to the five SNP difference thresholds were considered to be close genetically.

Analysis of population genetic structure was carried out with the hierBAPS (Bayesian analysis of population structure) hierarchical clustering algorithm implemented as rhierbaps R package (Cheng et al., 2013; Tonkin-Hill et al., 2018) on the core SNP alignment of the analysed isolates (with the NZ_CP016408.1 reference sequence removed). Clustering was performed with three hierarchical levels and 40 initial clusters with n.extra.rounds parameter set to 100,000,000 to ensure convergence of the algorithm.

In addition, the genomic relatedness amongst the 417 studied isolates was investigated with the hierarchical core genome multilocus sequence typing (cgMLST) clustering (HierCC) algorithm in EnteroBase (the “cgMLST V2 + HierCC V1” scheme; Zhou et al., 2020). A minimum spanning network (MSN) displaying cgMLST sequence types (STs) clustered at the HC0 hierarchical level was generated with MSTree V2 (Zhou et al., 2017). The MSN was annotated with the country of origin of the isolates, the presence of the IncFIB(pN55391) replicon, and the cluster identification numbers representing the clustering of the STs at the HC20 hierarchical level.

### 2.3 BEAST analysis of divergence times and origin of pESI or pESI-like megaplasmid

Bayesian Evolutionary Analysis Sampling Trees (BEAST v1.10.4) (Drummond and Rambaut, 2007) was used to perform a time-measured phylogenetic analysis to identify the likely origin of the pESI or pESI-like megaplasmid and explore substitution rates and divergence times of a subset of 417 *S.* Infantis isolates. The 245 isolates analysed with BEAST were selected to ensure maximum sampling (country of origin, host, sampling year, plasmid presence, and the identified AMR loci) and genomic trait (pairwise SNP distance of at least 25 SNPs, isolate placement on the ML tree) diversity (Supplementary Table S1). A core SNP alignment, computed as described above for the 245 sequences against the NZ_CP016408.1 reference, which comprised 3,864 SNPs, was used as input for BEAST.

The K3P (Kimura, 1981) substitution model with equal base frequencies was selected using ModelFinder (Kalyaanamoorthy et al., 2017) in IQTree v1.6.12 (Nguyen et al., 2015). Six combinations of molecular clock (strict vs. lognormal relaxed) and coalescent population size model (constant, logistic, or exponential) were run in BEAST. Each model combination was evaluated with at least two independent random-seed Monte Carlo Markov Chains (MCMCs) of a minimum of 400 million steps, with a sampling frequency set to collect 10,000 samples.

In all runs, a lognormal prior (mean = 3.4 × 10^−7^ [substitutions per site per year], standard deviation = 2, real parameter space) was used for the mean rate of the molecular clock, based on a mutation rate previously estimated for *S.* Typhimurium DT104 (Mather et al., 2013). A lognormal prior (μ = 10, σ = 2) was additionally used for mean population size. As the metadata for most isolates included only the sampling year and not a month or day, a uniform prior of 1 year was applied around all tip dates. As the alignment for BEAST comprised only polymorphic sites, the total number of nucleotides in the genome of the reference isolate (NZ_CP016408.1: 1,129,040, 1,235,773, 1,235,442, and 1,127,852, for A, C, G, and T, respectively) was incorporated in the xml file as an approximation of the non-variable sites in the analysis to avoid potential ascertainment bias.

Parameter mixing and convergence in MCMCs were verified by eye in Tracer v1.7.1 (Rambaut et al., 2018), as was an agreement between independent MCMC runs for each model combination. In all instances, 10% of samples were excluded as burn-in. Log files and tree files from independent MCMCs were combined using LogCombiner v1.10.4 (Drummond and Rambaut, 2007) and then re-verified in Tracer, confirming satisfactory effective sample sizes (> 200).

For each model combination, the log marginal likelihood was estimated with path sampling (PS)/stepping-stone sampling (SS), permitting alternative models to be compared through the calculation of Bayes factors (Baele et al., 2012, 2013). For each model combination, 100 PS/SS paths were used, with the number of iterations in each path equal to the number of MCMC iterations divided by 100. The combination of the lognormal relaxed molecular clock and logistic population size coalescent had the highest log marginal likelihood and was used for all subsequent analyses.

### 2.4 AMR gene and plasmid detection

Genomes of the analysed isolates were *de novo* assembled with shovill v.0.9.0^3^, with the depth and the gsize parameters set to 100 and 4.9 M, respectively. Subsequently, the assembled contigs of each isolate were scanned against the ResFinder (Zankari et al., 2012), PointFinder (Zankari et al., 2017), and PlasmidFinder (Carattoli et al., 2014) databases (versions from October 06, 2021, March 29, 2021, and July 12, 2021, respectively) with staramr v0.7.2 (Bharat et al., 2022) using default parameters. Specifically, the BLAST thresholds were as follows: minimum percent identity threshold between the top BLAST high-scoring pair (HSP) and the query sequence was set to 98; minimum percent length overlap threshold between the top BLAST HSP and the query sequence was set to 60 for the ResFinder and PlasmidFinder searches and 95 for the PointFinder searches. Staramr output tables detailing contigs containing sequences matching specific AMR genes, point mutations, and plasmid and replicon types were further processed and combined in R programming language. The gt^4^ R package was used to generate the final output table, which highlighted the contigs that matched both the AMR genes and point mutations and the plasmids, thus indicating whether the AMR genes and point mutations were harboured by particular plasmids. The predicted resistance phenotypes (antimicrobial class and compound) for the AMR genes detected with ResFinder were verified against a genotype-to-phenotype table downloaded from https://bitbucket.org/genomicepidemiology/resfinder_db/src/master/phenotypes.txt on 17.03.2022 and CARD (Alcock et al., 2020) web portal^5^, whereas for point mutations detected with PointFinder resistance phenotypes were verified against a genotype-to-phenotype table available at https://bitbucket.org/genomicepidemiology/pointfinder_db/src/master/salmonella/resistens-overview.txt (version from February 01, 2021). Resistance Gene Identifier v6.0.2 (Alcock et al., 2020) was used to distinguish between *qacE* and its truncated derivative *qacEdelta1*, both of which are disinfectant resistance genes (Kazama et al., 1998).

The presence of the pESI-like megaplasmid in the analysed isolates was additionally determined with SRST2 v0.2.0 (Inouye et al., 2014) by mapping WGS reads of each isolate to the reference which was the assembled sequence of pESI plasmid (RefSeq accession number: CP047882.1) from the Israel-isolated *S.* Infantis strain 119944 (Cohen et al., 2020).

IntegronFinder v2.0.2 (Néron et al., 2022) was used to detect integrons in the analysed isolates with the default parameters plus options local_max and func_annot. The software was applied to the 417 assembled genomes of isolates sequenced with the short-read sequencing technology and to the hybrid genome assemblies of 10 isolates that were also sequenced with the long-read sequencing technology (see below).

### 2.5 Long-read sequencing

To more accurately characterise the presence and localisation of the AMR genes and plasmids in the analysed *S.* Infantis isolates, long-read sequencing was performed on 10 selected APHA-sequenced *S.* Infantis isolates (Supplementary Table S7) on a MinION flow cell (Oxford Nanopore Technologies) with the Rapid Barcoding Sequencing (SQK-RBK004) kit following the manufacturer’s instructions. The near real-time basecalling of the sequenced genomes was implemented via the Fast model of the Guppy basecaller algorithm. A hybrid genome assembly comprising both the Nanopore long reads and the Illumina short reads was constructed for each of the 10 isolates using Unicycler v0.4.9b (Wick et al., 2017). The software was run using the default parameters. The staramr pipeline was applied to the hybrid genome assemblies as described above.

## 3 Results

### 3.1 Genetic clustering at the wide geographical scale

**Figure 1:**
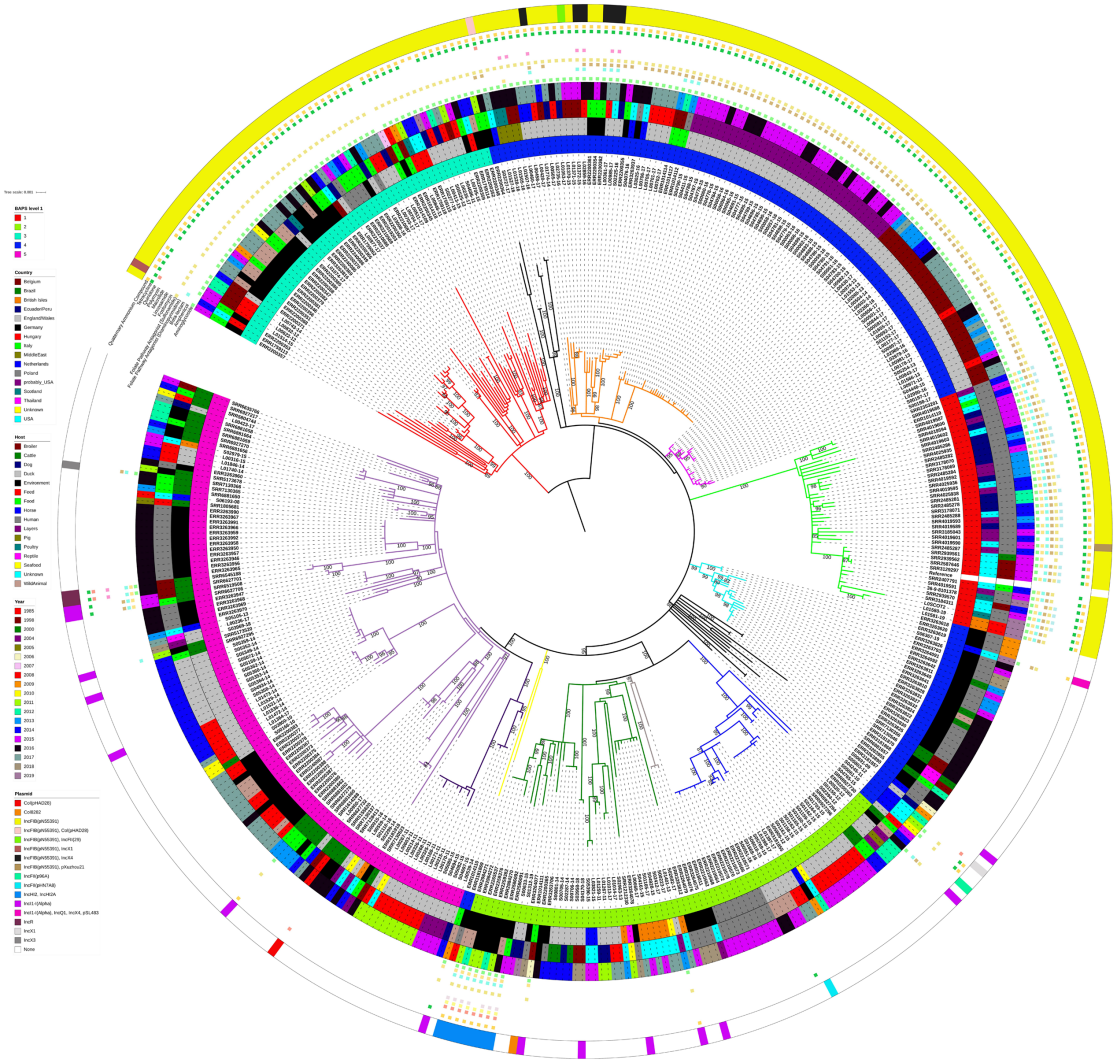
Midpoint-rooted maximum-likelihood phylogenetic tree with 415 isolates from the large geographical scale panel of *S.* Infantis isolates. The phylogeny is based on core polymorphic SNPs derived from a multisequence alignment. Main clades are differentiated by coloured branches. The innermost annotation ring specifies the hierBAPS cluster each isolate was assigned to, the second annotation ring specifies the country of origin, the third annotation ring specifies the host, the fourth annotation ring specifies the year of isolation, and the outermost ring specifies the detected plasmids (or plasmid replicon types). Resistance profiles to specific antimicrobial classes are represented by coloured squares. See Supplementary Table S5 for details on which AMR genes conferred resistance to which antimicrobial classes and compounds. Bootstrap branch support values between 80% and 100% are shown on the tree.

There was a clear pattern of the 194 *S.* Infantis isolates harbouring replicon IncFIB(pN55391), typical of the pESI or pESI-like megaplasmids, clustering together and separately from the isolates that lacked the IncFIB(pN55391) replicon. This pattern of the large geographical scale panel of *S.* Infantis isolates clustering with respect to the presence/absence of the IncFIB(pN55391) replicon rather than with respect to the country of origin of an isolate, the host, or the year of isolation was indicated by the ML phylogenetic tree and the hierBAPS algorithm based on the core genome MSA (Figure 1), as well as by the cgMLST-based minimum spanning network (cgMLST analysis is briefly described below; for additional details, see Supplementary Text, Supplementary Figure S1, Supplementary Table S4).

hierBAPS split the 415 isolates into five genetic clusters at the first hierarchical level, 21 genetic clusters at the second hierarchical level, and 67 genetic clusters at the third hierarchical level. At the first hierarchical level, hierBAPS clustering was broadly congruent with the topology of the ML tree (Figure 1). Two of the five hierBAPS first hierarchical level clusters, namely, clusters 1 and 3, comprised exclusively IncFIB(pN55391) harbouring isolates (some also with another plasmid replicon type or types). hierBAPS cluster 1 (red colour, Figure 1), which overlapped with the light green ML tree clade, was made up of 13 isolates from the USA (including seven isolates from humans and four from broilers), 22 isolates from USA patients with no data on their travel history (“probably USA” samples) or with travel history to Ecuador/Peru, 2 isolates from the British Isles (from an unknown source), 1 Italian isolate sampled from a human, and 2 Scottish isolates (both were human isolates) (Figure 1). These isolates were characterised by genes conferring resistance to aminoglycosides (*aac(3)-IV*, *ant(3″)-Ia*, *aph(3′)-Ia*, *aph(4)-Ia*), amphenicols (*floR*), beta-lactams (*bla*_CTX-M-65_), diaminopyrimidines (*dfrA14*), sulfonamides (*sul1*), fosfomycins (*fosA3*), tetracyclines (*tet*(A)), and quaternary ammonium compounds (*qacEdelta1*) (Figure 1; Supplementary Table S5). All hierBAPS cluster 1 isolates were detected to harbour the *gyrA* (D87Y) and *parC* (T57S) chromosomal point mutations (resistance to nalidixic acid and ciprofloxacin) (Supplementary Table S5). hierBAPS cluster 3 (cyan colour, Figure 1), which overlapped with the red ML tree clade, comprised 55 *S.* Infantis isolates originating from six European countries: 1 isolate from Belgium, 13 E/W isolates, 26 German isolates, 8 Hungarian isolates, 2 isolates from Italy, and 5 isolates from Poland (Figure 1). Of these, 25 isolates were from animal hosts, 24 originated from samples from the environment, animal feed, or food produce, 5 were from unknown sources, and a single isolate was from a human. A total of 50 of these isolates were obtained from samples taken after 2011. The majority of the hierBAPS cluster 3 isolates harboured the *ant(3″)-Ia*, *sul1*, *tet*(A), and *qacEdelta1* AMR and disinfectant resistance genes. One hierBAPS cluster 3 isolate from Hungary was detected to harbour genes conferring resistance to beta-lactams (*bla*_TEM-1B_) and quinolones (*qnrS1*) but not to aminoglycosides, sulfonamides, or quaternary ammonium compounds (Figure 1; Supplementary Table S5). All hierBAPS cluster 3 isolates harboured the *gyrA* (S83Y) and *parC* (T57S) chromosomal point mutations (resistance to nalidixic acid and ciprofloxacin) (Supplementary Table S5).

hierBAPS cluster 4 (blue colour, Figure 1), which was polyphyletic, comprised 99 isolates that harboured the IncFIB(pN55391) replicon and 37 isolates in which this plasmid replicon type was not detected (Figure 1). The 99 IncFIB(pN55391) harbouring isolates occupied the neighbouring black, orange, and pink ML tree clades (Figure 1). Of the 99 isolates, 83 were from E/W, the vast majority of which were from broiler farms A, B, and C, processing plant D, and layer farm E, sampled between 2013 and 2017. Isolates from this cluster also originated from Germany (six isolates), Italy (three isolates), the Middle East (four isolates), and the Netherlands (three isolates). Just two of these 99 isolates were from human hosts: one isolate from Germany and one isolate from Italy. Resistance genes detected in the 99 hierBAPS cluster 4 isolates that harboured IncFIB(pN55391) comprised *aac(3)-IV*, *aadA2*, *aadA22*, *ant(3″)-Ia*, *aph(3′)-Ia*, and *aph(4)-Ia* (aminoglycoside resistance), *floR* (amphenicol resistance), *bla*_CTX-M-1_ and *bla*_TEM-1B_ (beta-lactam resistance), *dfrA1* and *dfrA14* (diaminopyrimidine resistance), *sul1* and *sul2* (sulfonamide resistance), *lnu*(F) and *lnu*(G) (lincosamide resistance), *qnrB19* (quinolone resistance), *tet*(A) (tetracycline resistance), and *qacEdelta1* (quaternary ammonium compound resistance) (Figure 1; Supplementary Table S5). Chromosomal point mutations in *gyrA* (D87G), *gyrA* (D87Y), *gyrA* (S83Y), and *parC* (T57S) (resistance to nalidixic acid and ciprofloxacin) were detected in the hierBAPS cluster 4 IncFIB(pN55391) harbouring isolates (Supplementary Table S5).

In addition to the 37 hierBAPS cluster 4 isolates that lacked the IncFIB(pN55391) replicon, all hierBAPS cluster 2 (75 isolates, light green colour in Figure 1) and hierBAPS cluster 5 (109 isolates, pink colour in Figure 1) isolates lacked this plasmid replicon type. The 221 isolates without IncFIB(pN55391) clustered together in several distinct clades on the ML tree. These isolates were mostly from Europe (76 from Germany, 75 from E/W, 10 from Holland, 9 from the British Isles, 9 from Poland, 2 from Italy, and 4 from an unknown locality likely from within the European Union), plus one isolate from Thailand, and 35 isolates from Brazil (i.e., all analysed Brazilian isolates in this study, Table 1) that were interspersed amongst the European isolates on the phylogenetic tree (Figure 1). The majority (33) of the E/W isolates without the IncFIB(pN55391) replicon were from animal feed, whereas the majority of German isolates (44) were from humans. Of the 221 isolates without the IncFIB(pN55391) replicon, there were 31 that were carrying another plasmid replicon type or types. Eight German isolates, harbouring the IncHI2 and IncHI2A plasmids and carrying genes conferring aminoglycoside resistance: *aac(6′)-Ib3*, *aadA1* (six isolates) or *aadA1b* (two isolates), *aph(3″)-Ib*, and *aph(6)-Id*; amphenicol resistance: *catA1*; beta-lactam resistance: *bla*_ACC-1_ and *bla*_VIM-1_; sulfonamide resistance: *sul1*; macrolide resistance: *ere*(A) (seven isolates); polymyxin resistance: *mcr-9*; quinolone resistance: *aac(6′)-Ib-cr*; and quaternary ammonium compound resistance: *qacEdelta1*, clustered together in the dark green clade of the ML tree, whereas isolates harbouring other plasmid replicon types were evenly distributed throughout the different clades of the ML tree (Figure 1). Many of the isolates carrying those plasmids also harboured one or more AMR genes (Figure 1; Supplementary Table S5).

EnteroBase grouped the 417 *S.* Infantis isolates into 379 STs in accordance with their cgMLST allele profiles that comprised 75 distinct clusters at the HC20 hierarchical level (Supplementary Figure S1; Supplementary Table S4). The structure of the 75 HC20 hierarchical level clusters (Supplementary Table S4) was highly congruent with how the isolates were assigned to hierBAPS clusters and phylogenetic clades on the ML tree (Figure 1). At the HC50 hierarchical level, there were seven distinct clusters, whereas at the HC100 hierarchical level, only two distinct clusters were formed, one with all the 415 isolates presented on the ML tree in Figure 1 and the other with the two highly genetically distinct isolates from the Netherlands: L01384-14 and S05257-14 (Supplementary Table S4). The minimum spanning network (MSN) was composed of 352 nodes, with each node representing a single HC0 cluster, to which the 417 analysed isolates were assigned (Supplementary Figure S1; Supplementary Table S4). Node size was proportional to the number of isolates belonging to each HC0 cluster. The topology of the MSN was broadly congruent with the topology of the 415 isolate ML tree (Figure 1), particularly concerning the relative placement of isolates from the different localities of origin and isolates with and without the pESI or pESI-like megaplasmid.

The maximum pairwise SNP distance was 174 SNPs between a German isolate, ERR2200388, from animal feed and an E/W isolate, L00511-17, from a dog (Supplementary Table S2). The second highest SNP distance of 173 SNPs was between L00511-17 and a German isolate, ERR2240087, sampled from the environment (SNP distance between ERR2200388 and ERR2240087 was five SNPs). L00511-17, which harboured the IncFIB(pN55391) replicon, was assigned to hierBAPS cluster 3 and was situated on the deepest branch of the red ML tree clade, whereas ERR2240087 and ERR2200388 belonged to hierBAPS cluster 5 and the light violet ML tree clade and both lacked IncFIB(pN55391). Isolate L00511-17 was also highly differentiated from all other isolates that were part of the 415 isolate ML tree according to the SNP address as this isolate shared an SNP address with the other 414 isolates only at the highest SNP address threshold of 250 SNPs (Supplementary Table S3). The two Europe-wide ring trial isolates from the Netherlands (L01384-14 and S05257-14) that were excluded from the ML tree exhibited an SNP address threshold in excess of 250 SNPs from all other isolates (Supplementary Table S3). Based on the short-read sequencing, these two isolates did not carry any plasmids but had the chromosomal *parC* (T57S) point mutation. Isolates that shared SNP address up to the 50 SNP threshold (i.e., the first three numbers of the 7-digit SNP address) were always assigned to the same hierBAPS cluster and, with the exception of SNP address 1.1.3.x.x.x.x, always grouped together in the same ML tree clade (Supplementary Table S3). All 106 isolates with the SNP address 1.1.3.x.x.x.x were assigned to the split hierBAPS cluster 4, and hence, this group of isolates comprised isolates both with and without the IncFIB(pN55391) replicon.

### 3.2 English and Welsh isolates

**Figure 2:**
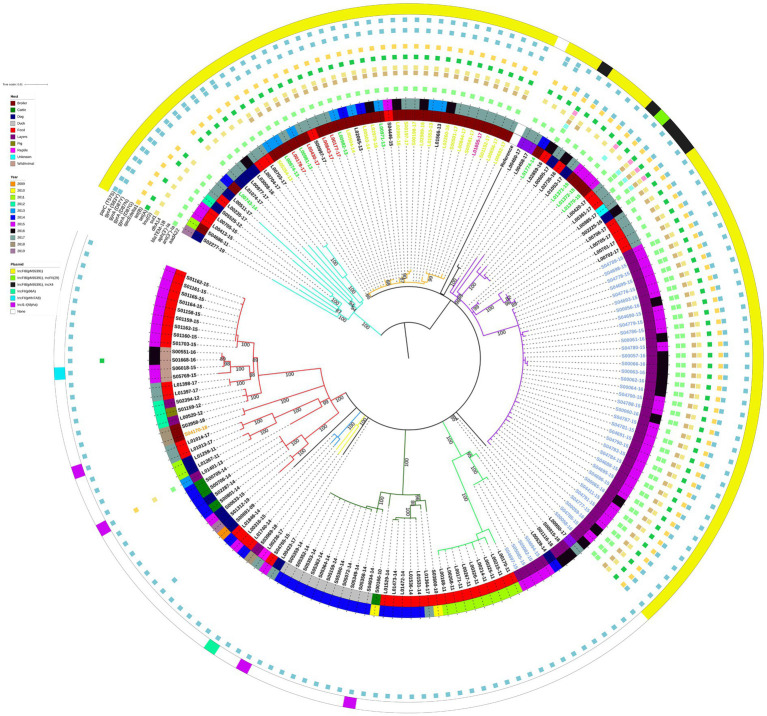
Midpoint-rooted maximum-likelihood phylogenetic tree with 171 isolates from the English and Welsh panel of *S.* Infantis isolates. The phylogeny is based on core polymorphic SNPs derived from a multisequence alignment. Main clades are differentiated by coloured branches. The innermost annotation ring specifies the host of the analysed isolates, the middle annotation ring specifies the year of isolation, and the outermost annotation ring specifies the detected plasmids (or plasmid replicon types). Detected antimicrobial resistance genes and point mutations are represented by coloured squares. See Supplementary Table S5 for details on which AMR genes conferred resistance to which antimicrobial classes and compounds. Labels of 16 isolates from farm A (broiler farm) are on yellow-green background, labels of 4 isolates from farm B (broiler farm) are on red background, label of a single isolate from farm C (broiler farm) is on pink background, labels of 9 isolates from processing plant D are on green background, labels of 40 isolates from farm E (laying farm) are on blue background, and label of a single isolate from farm F (broiler farm—non-AMR strain) is on an orange background. Bootstrap branch support values between 80% and 100% are shown on the tree.

Similarly to the isolate clustering patterns evident on the large geographical scale phylogeny, the E/W isolates with the IncFIB(pN55391) replicon clustered separately from the E/W isolates without this replicon type on the ML tree constructed with 171 E/W *S.* Infantis isolates only (Figure 2). Most of the E/W isolates with IncFIB(pN55391) were from samples taken between 2015 and 2017, whereas a large proportion of the E/W isolates without IncFIB(pN55391) were from 2011, 2014, and 2015. There was some evidence of isolates originating from a single year or subsequent years clustering together on the ML tree, as several clades, including the purple (2015–2016), red (2015), dark green (2014), and light green (2011) ML tree clades grouped substantial numbers of isolates with the same year of isolation, although many of the isolates with the same year of isolation were collected from the same premise (Figure 2).

Similarly, clustering by host was visible only in certain clades of the ML tree. The red, dark green, and light green ML tree clades that comprised isolates lacking the IncFIB(pN55391) replicon were mainly animal feed isolates, and all of the duck isolates (all from the same premise) clustered in the dark green clade (Figure 2). For the IncFIB(pN55391) harbouring isolates, most of the isolates from the orange clade were from broilers, and the majority of the violet clade isolates were from layers sampled on farm E (Figure 2). The shallow branching amongst groups of isolates comprising some of the ML tree clades, such as violet clade farm E layers isolates from 2015 and 2016 or dark green clade duck isolates from 2014 (Figure 2), suggested a clonal or nearly clonal nature of these groups of isolates. This conclusion was supported as the SNP addresses of those isolates were identical up to the 5 SNP threshold (Supplementary Table S3), indicating low within-premise and between-year genetic diversity at some of the sampled premises.

Almost all of the E/W poultry isolates from broiler farms A, B, and C, from poultry processing plant D, and from laying farm E clustered in two neighbouring clades of the phylogenetic tree, i.e., the orange and violet ML tree clades, suggesting substantial levels of genetic relatedness amongst isolates sampled from these five E/W premises of epidemiological interest (Figure 2). This was supported by the average pairwise SNP distance for this group of isolates of 19.8 SNPs (Supplementary Table S2). The IncFIB(pN55391) replicon was detected in isolates collected from all five premises (Figure 2). Four out of 40 isolates from laying farm E were found not to harbour any plasmid replicon types, but all isolates from broiler farms A, B, and C, and from poultry processing plant D, harboured IncFIB(pN55391), some also with one other plasmid replicon type. The single isolate from broiler farm F harboured IncI1-I(Alpha) (Figure 2). For many of the isolates from premises of interest A, B, C, and D, the resistance gene profile comprised *ant(3″)-Ia*, *dfrA14*, *sul1*, *tet*(A), and *qacEdelta1*. The majority of the IncFIB(pN55391) harbouring isolates from laying farm E also carried *aph(3′)-Ia* (Figure 2). *bla*_TEM-1B_ was detected in a single isolate from poultry processing plant D.

### 3.3 Time-measured phylogeny and phylogeography of IncFIB(pN55391)-carrying *S.* Infantis isolates

**Figure 3:**
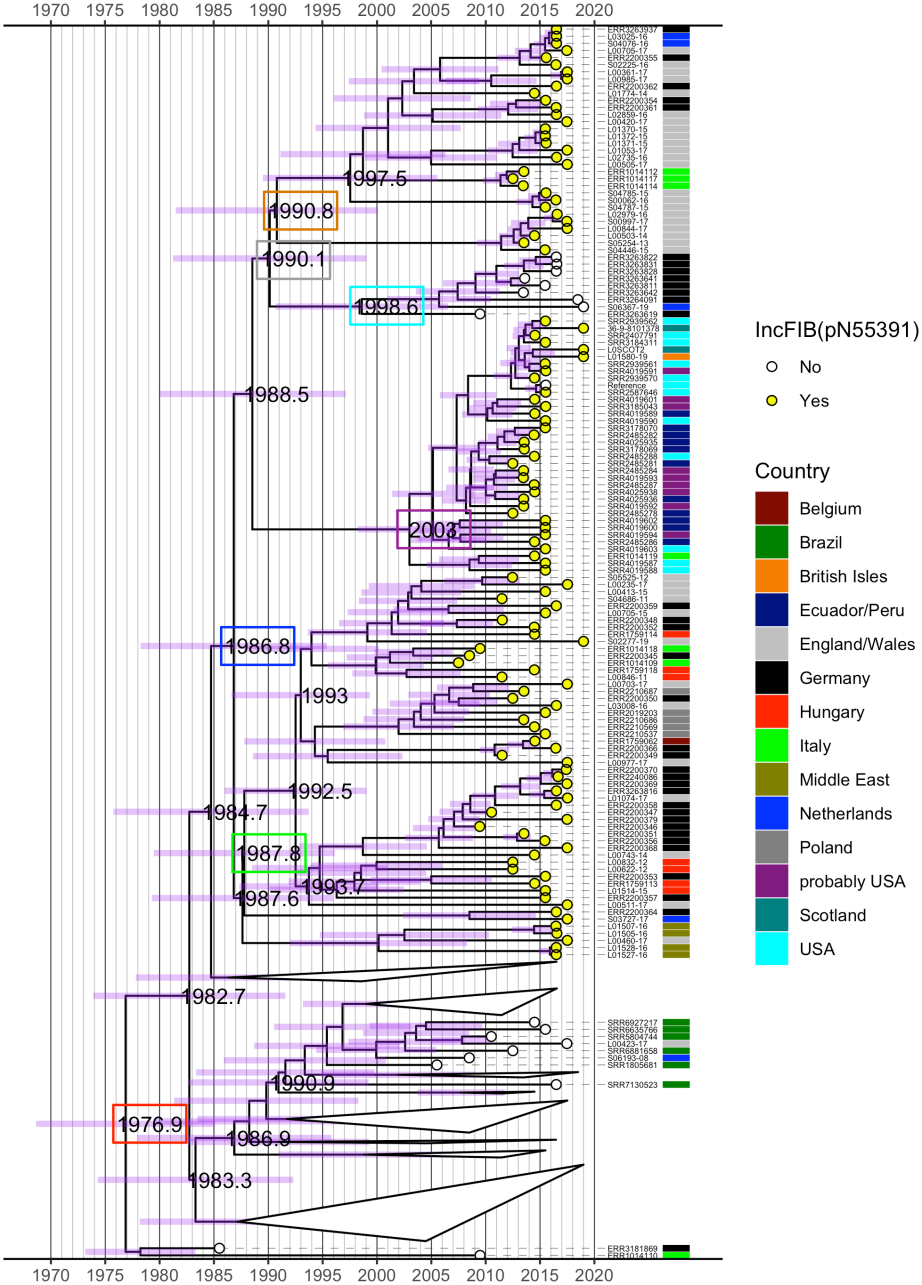
Time-measured maximum clade credibility (MCC) tree from BEAST for 245 *S.* Infantis isolates. Years of the occurrence of key evolutionary events are specified on the tree and 95% height posterior density (HPD) is represented by purple bars for all phylogenetic splits. Selected dates are shown inside coloured boxes. See text for details. The tree is annotated by the country of origin of the analysed isolates and the presence (yellow circle) or absence (white circle) of the IncFIB(pN55391) replicon representative of the pESI or pESI-like megaplasmid. Some clades in the bottom half of the tree that featured exclusively isolates without IncFIB(pN55391) were collapsed for clarity. See Supplementary Figure S2 for the full MCC tree.

The time-measured maximum clade credibility (MCC) tree from BEAST revealed that the most recent common ancestor (MRCA) of all analysed isolates was dated to ~1977 (95% height posterior density (HPD) 1968–1985) (red box, Figure 3; Supplementary Figure S2). Visual inspection of the MCC tree and the 415 isolate ML phylogeny (Figure 1) showed strong agreement in the topology with regard to fine-scale relationships between isolates and the macro-clade structure split by the presence/absence of the IncFIB(pN55391) replicon and the isolate’s country of origin.

As with the ML phylogeny, the isolates that possessed the IncFIB(pN55391) replicon formed a single distinct clade (descended from a single branch), which diverged from isolates without IncFIB(pN55391). The MRCA of isolates with IncFIB(pN55391) was estimated at 1987 (95% HPD 1978–1995) (blue box, Figure 3; Supplementary Figure S2). Within this monophyletic group, with the exception of a basal branch leading to four Middle East and one E/W isolate, the deepest bifurcation led to a large clade of European isolates with a crown age ~ 1988 (95% HPD 1979–1996) (green box, Figure 3; Supplementary Figure S2). The oldest splits within this European clade suggested preliminary radiation in E/W (isolates belonging to the red ML tree clade and hierBAPS cluster 3 in Figure 1), Germany, Hungary, and the Netherlands, followed by spread through Belgium, Poland, and Italy, occurring between ~1992 and 2000. In comparison, a distinct clade of IncFIB(pN55391) possessing isolates of predominantly USA origin and USA patients with a travel history to South America origin was younger, with an MRCA dated to ~2003 (95% HPD 1998–2010) (purple box, Figure 3; Supplementary Figure S2), and the bulk of divergences having taken place between 2005 and 2011. Thus, overall, the time-measured BEAST tree indicated that for the analysed isolates, the initial acquisition of the IncFIB(pN55391) plasmid replicon type likely occurred in Europe in the late 1980s, with a single introduction of IncFIB(pN55391)-carrying *S.* Infantis isolates to the Americas at some point after this. A second incursion of the IncFIB(pN55391)-carrying isolates occurred in E/W after 1991 (95% HPD 1981–2000) (orange box, Figure 3; Supplementary Figure S2). This clade included the majority of the BEAST-analysed E/W IncFIB(pN55391) possessing isolates and several isolates each from Germany, Italy, and the Netherlands (isolates which belonged to the orange and pink ML tree clades and hierBAPS cluster 4 in Figure 1). Within this secondary European clade, short tip branches suggest substantial recent divergences between 2011 and 2015 (Figure 3; Supplementary Figure S2).

Nested within the isolates with the IncFIB(pN55391) plasmid replicon type was a clade of nine isolates for which IncFIB(pN55391) was not detected (isolates that belonged to the blue ML tree clade and hierBAPS cluster 4 in Figure 1). Eight of these nine, including the most basal, were from Germany, and together, they had a crown age of 1999 (95% HPD 1991–2006) (cyan box, Figure 3; Supplementary Figure S2) and diverged from their sister clade, the secondary younger clade of IncFIB(pN55391)-possessing European isolates, in 1990 (95% HPD 1981–1999) (grey box, Figure 3; Supplementary Figure S2). Thus, in contrast to the ML tree, the Bayesian topology showed evidence of pESI or pESI-like megaplasmid loss (Figure 3; Supplementary Figure S2).

### 3.4 Plasmid presence and antimicrobial resistance genes

Short-read genome assembly-based *in silico* detection and typing of plasmids revealed that 53.94% of the 417 analysed isolates carried at least one plasmid (Supplementary Table S5). A total of 16 different plasmid types were identified in the 225 plasmid-carrying isolates of which the most common was the IncFIB(pN55391) plasmid replicon type that was harboured by 86.22% of these isolates (Supplementary Table S5). Of the 225 isolates, 18 harboured two distinct plasmids, and a single isolate (isolate S06367-19 from the Netherlands that was part of Europe-wide ring trials) harboured four distinct plasmids. Almost all isolates that carried a plasmid, in particular, the IncFIB(pN55391) replicon, were also detected to harbour one or more AMR genes (excluding point mutations but including *qacEdelta1*). Of the 194 isolates that harboured IncFIB(pN55391), there were no AMR genes detected in only two isolates. Of the 31 isolates found to harbour plasmid replicon type or types other than IncFIB(pN55391), nine isolates did not have any AMR genes identified. In contrast, of the 192 isolates in which no plasmids were found, just five isolates harboured AMR genes (three isolates with a single AMR gene each (*aac(6′)-Iaa* in two isolates and *tet*(B) in a single isolate) and two isolates with six AMR genes each: *ant(3″)-Ia*, *aph(3″)-Ib*, *aph(6)-Id*, *sul1*, *tet*(A), and *qacEdelta1* (Supplementary Table S5)). However, a demonstration that in the plasmid-carrying isolates the identified AMR genes were associated with the detected plasmids was difficult to establish based on the short-read sequencing data. Just 18 out of a total of 246 identified plasmid replicon types were detected on the same contig as an AMR gene or genes. For IncFIB(pN55391), out of the 194 isolates that had this replicon type, only in isolate ERR2200355 the detected AMR gene (*dfrA14*) was present on the same contig as the IncFIB(pN55391) replicon. Plasmid replicon types other than IncFIB(pN55391) that were detected in at least three isolates included IncX4, which was detected in seven isolates (in six isolates together with IncFIB(pN55391) and in one isolate together with IncI1-I(Alpha), IncQ1, and pSL483) and was found to carry a resistance gene or genes in six of these isolates. In addition, IncI1-I(Alpha), which was detected in a total of 15 isolates, was found to carry a resistance gene or genes in six of these isolates (Supplementary Table S5).

For all 194 isolates that were found to possess the IncFIB(pN55391) replicon, SRST2 indicated the presence of the CP047882 reference sequence (pESI plasmid from the Israel-isolated *S.* Infantis strain 119944) at minimum 77.95% identity (in an E/W isolate), and for 178 of these isolates, the sequence homology exceeded 95.00%.

In all, 43 different resistance genes (conferring resistance to 12 antimicrobial classes) and five core genome point mutations (a single resistance phenotype) were detected *in silico* in the 417 *S.* Infantis isolates (Supplementary Table S5). Seven isolates (all from Germany) harboured 12 resistance genes, and 16 isolates (14 from USA with the travel history, 1 from Germany, and 1 from Italy) harboured 11 resistance genes (Supplementary Table S5). Eight of these 23 isolates were carrying *bla*_ACC-1_ and the remainder were carrying *bla*_CTX-M-65_. A single isolate (SRR7130366, from Brazil and isolated from food) was found to carry *bla*_TEM-1A_ (associated with IncX3 plasmid), and 11 isolates were carrying *bla*_TEM-1B_. One of these isolates was from Hungary (*bla*_TEM-1B_ associated with IncX1 plasmid), one from Germany (*bla*_TEM-1B_ associated with IncX4 plasmid), five from E/W (*bla*_TEM-1B_ associated with IncX4 plasmid), and four from Brazil (no detected association between *bla*_TEM-1B_ and a plasmid). *sul1* was the most common resistance gene identified in the analysed isolate dataset (204/417 isolates), and this was followed by *qacEdelta1* (203/417), *tet*(A) (197/417), *ant(3″)-Ia* (189/417), and *dfrA14* (124/417) (Supplementary Table S5). Across the whole dataset, there were 612 point mutations that were detected in two genes: *gyrA* (point mutation at four nucleotide positions) and *parC* (point mutation at a single-nucleotide position). Point mutations in both of these chromosomal genes were associated with resistance to nalidixic acid and ciprofloxacin. The *parC* point mutation was detected in all of the 417 analysed isolates, and one of the four *gyrA* point mutations (always a single *gyrA* point mutation detected per isolate) was detected in 195 isolates (Supplementary Table S5).

IntegronFinder version 2 detected the presence of “complete” (detection of integron integrase (IntI) and nearby *attC* site(s)) class I integrons in 196 isolates (Supplementary Table S6A) and of “CALIN” (detection of *attC* sites(s) only) class I integrons in 21 isolates (Supplementary Table S6B). In addition, there were also 15 detections of “In0” elements (detection of IntI only) for class I integrons. Eight isolates were detected to harbour complete class II integrons, but these only contained IntI and a single *attC* site each, and no other genes were detected on any class II integrons (Supplementary Table S6C).

A total of 112 of the 196 isolates that were detected to harbour complete class I integrons contained a set of two integrons (IntI detected on two separate contigs) (Supplementary Table S6A). Just eight isolates detected to carry complete class I integron(s) lacked the pESI or pESI-like megaplasmid, but five of those isolates harboured another type of plasmid or plasmids. Conversely, six isolates found to carry the pESI or pESI-like megaplasmid were not detected to harbour any complete class I integrons. There were only a few cases where the plasmid replicon and IntI of a complete class I integron were present on the same contig, confirming that the integron was part of a plasmid: a single occurrence for IncFIB(pN55391) and another one for IncQ1 and four occurrences for IncI1-I(Alpha). Gene content of complete class I integrons comprised *aadA1* (184 isolates), *ant(3″)* (seven isolates), *cmlA* (amphenicol resistance; two isolates), *dfrA* (110 isolates), *qac* (two isolates), and *qacE* (189 isolates) (Supplementary Table S6A).

Out of the 22 isolates that were detected to contain CALIN class I integrons, 12 harboured the pESI or pESI-like megaplasmid, eight the IncHI2 and IncHI2A plasmids, and one isolate carried the IncX3 plasmid. *aac(6′)-Ib* (eight isolates), *aadA1* (two isolates), *ant(3″)* (two isolates), *blaVIM* (eight isolates), *lnu*(F) (two isolates), *dfrA* (nine isolates), and *sat2* (nucleoside resistance; one isolate) were the genes detected on CALIN class I integrons (Supplementary Table S6B).

### 3.5 Hybrid assembly AMR gene and plasmid presence and localisation

The 10 hybrid genome assemblies were composed of 3 to 28 contigs per isolate, and the assembled contigs were circularised in 3 of the 10 isolates (Supplementary Table S7A). Nonetheless, for every one of the 10 hybrid assemblies, it was possible to deduce which contigs represented the chromosome and which contigs represented the largest of the detected plasmids: the pESI or pESI-like megaplasmid harbouring the IncFIB(pN55391) plasmid replicon type (Supplementary Table S7A).

The presence of IncFIB(pN55391) and other plasmid replicon types in the hybrid genomes of the 10 isolates was indicated by staramr applied to the hybrid assemblies (Supplementary Table S7B). For these 10 isolates, exactly the same plasmids were identified based on the short-read assemblies (Supplementary Table S5) and the hybrid assemblies (Supplementary Table S7B). This was also true regarding the detected AMR genes but with a few exceptions. Based on the short-read assembly staramr output, the aminoglycoside resistance gene *aadA22* was present in isolates L00985-17 and L02859-16 (Supplementary Table S5), but based on the hybrid assembly staramr output, this gene was not detected in these two isolates but was found to be present in isolate L00420-17 (Supplementary Table S7B). Another aminoglycoside resistance gene, *ant(3″)-Ia*, was found to be present in isolates L00985-17 and L02859-16 based on the hybrid assemblies only (Supplementary Table S7B).

The major difference between staramr outputs based on the short-read and hybrid assemblies for these 10 isolates was the localisation of the AMR genes. While staramr analysis based on the short-read assemblies showed that 7.72% of the detected plasmid replicon types were located on the same contig as an AMR gene or genes (Supplementary Table S5), the hybrid assemblies of the 10 isolates indicated that 88.89% of the detected plasmid replicon types were located on the same contig as an AMR gene or genes (Supplementary Table S7B). The pESI or pESI-like megaplasmid (red contig label), detected in 8 of the 10 isolates, harboured the *ant(3″)-Ia*, *aph(3′)-Ia*, *sul1*, *tet*(A), *dfrA14*, and *qacEdelta1* AMR genes. Plasmid Col(pHAD28) (blue contig label), detected in a single isolate (S03727-17), harboured *qnrB19*. The *qnrB19* gene was also detected on plasmid IncX1 (orange contig label) in isolate S05803-12. Plasmids IncI1-I(Alpha) and IncQ1 (black contig label), both present on the same contig in isolate S06367-19, harboured seven different AMR genes (Supplementary Table S7B). The pESI or pESI-like megaplasmid was not detected in this isolate, and some of the AMR genes harboured by IncI1-I(Alpha) and IncQ1 in S06367-19 were detected on the same contig as IncFIB(pN55391) in the isolates that harboured this plasmid replicon type. IncX4 was detected in five isolates but was associated with AMR genes in only four (brown contig label). The IncX4 plasmid carried *bla*_TEM-1B_ in these four isolates and *lnu*(G) in three of these isolates. Plasmid pSL483 was detected in isolate S06367-19, but it was not associated with any AMR genes. The only AMR genes that were not associated with any plasmids according to this analysis were *lnu*(G) and *aadA22,* detected on contig07 in isolate L00420-17, and eight genes detected on contig02, which at 1,731,284 bases long was a core genome contig (Supplementary Table S7A), in isolate L01505-16 (Supplementary Table S7B). Chromosomal point mutations that conferred resistance to nalidixic acid and ciprofloxacin were detected in *gyrA* (point mutation at three nucleotide positions) and *parC* (point mutation at a single-nucleotide position) (Supplementary Table S7B).

Nine of the ten Nanopore sequenced isolates were detected to carry complete class I integrons (four isolates harboured IntI integrases on two different contigs) (Supplementary Table S6D), and six of those isolates were also detected to carry complete class II integrons (a single IntI integrase was detected in all six isolates) (Supplementary Table S6E), always on the same contig that harboured a class I integron IntI integrase. There was also a single detection of the In0 element for a class I integron. For the isolates that harboured the pESI or pESI-like megaplasmid, the IncFIB(pN55391) replicon was always present on the same contig as the complete class I and class II integron IntI integrases. Other plasmids detected to contain complete class I integrons were IncI1-I(Alpha), IncQ1, and IncX4. The identified complete class I integron genes comprised *aadA1* (seven isolates), *ant(3″)* (four isolates), *dfrA* (two isolates), *lnu*(F) (one isolate), *lnu*(G) (three isolates), and *qacE* (eight isolates) (Supplementary Table S6D). For the complete class II integrons, the identified genes were *aadA1* (one isolate), *dfrA* (five isolates), and *qacE* (one isolate) (Supplementary Table S6E).

## 4 Discussion

The genetic population structure was investigated of 415 *Salmonella enterica* subsp. *enterica* serovar Infantis isolates from diverse geographical locations with a particular focus on Europe, North America, and South America. Utilising the WGS data of this set of isolates, several different clustering analyses were carried out, including ML phylogenies and the hierBAPS algorithm, both based on the polymorphic SNPs derived from a multisequence alignment, as well as a minimum spanning network based on the hierarchical clustering of cgMLST alleles. Overall, there was a general congruence in how these different clustering methods grouped the analysed isolates. In particular, it was clear that, with the exception of the isolates originating from the USA and those obtained from USA salmonellosis patients with travel history to Latin America, there was limited clustering of isolates by their country of origin. Interestingly, 35 Brazilian *S.* Infantis isolates grouped on the large geographical scale ML tree amongst the isolates of European origin, and rather than forming their own distinct clade, were nested within two clades that otherwise largely comprised the E/W, German, and Polish isolates. Moreover, the analysed isolates visibly did not cluster by their source of origin. This result corroborated the recent studies of Gymoese et al. (2019) and Alba et al. (2020) who did not detect an obvious correlation between the genetic clustering and the source or the geographical origin of the isolates, which might be indicative of global dissemination of some MDR strains (Bogomazova et al., 2020; Mattock, 2020; Nagy et al., 2020; Kürekci et al., 2021). However, our results showed that there was a clear clustering of isolates that were found to carry the IncFIB(pN55391) replicon, which was indicative of isolates harbouring a pESI or pESI-like megaplasmid. On the large geographical scale ML tree, the pESI or pESI-like megaplasmid harbouring isolates belonged exclusively to the initial (from left to right) six neighbouring phylogenetic clades. hierBAPS-derived genetic clusters did not completely overlap with the ML tree in this regard, as of the three hierBAPS clusters overlapping with the above-specified ML tree clades, the polyphyletic hierBAPS cluster 4 also included 37 isolates that lacked the IncFIB(pN55391) replicon. At the second hierarchical level, hierBAPS cluster 4 was split further into four clusters. Two of these clusters comprised isolates with and without the IncFIB(pN55391) replicon. The cgMLST-based hierarchical clustering also supported clustering of the isolates based on the presence/absence of the IncFIB(pN55391) replicon as multiple clusters at the HC20 level, including clusters number: 343, 1369, 1272, and 7828, were made up exclusively of isolates that had this plasmid replicon type. Studies by Franco et al. (2015), Brown et al. (2018), Gymoese et al. (2019), Alba et al. (2020), García-Soto et al. (2020), and Lee et al. (2021) also largely supported the conclusion of genetic clustering of the studied isolates in accordance with the presence or absence of the pESI or pESI-like megaplasmid.

Clustering of the IncFIB(pN55391) replicon harbouring isolates, many of which were of diverse geographical origins, was also evident on the time-measured phylogeny. A highly interesting outcome of the BEAST analysis was an indication that the most recent common ancestor of the analysed set of isolates was dated to ~1977. The basal clade of the time-measured phylogeny comprised German isolate ERR3181869, obtained from a human in 1985, and Italian isolate ERR1014110, which was isolated from a wild animal in 2009. Both isolates were part of the same clade on the large geographical scale ML phylogeny and were assigned to hierBAPS cluster 4. Crucially, according to the MCC tree from BEAST, the most recent common ancestor of the pESI or pESI-like megaplasmid harbouring isolates was dated to ~1987 (having originated likely somewhere in Europe), thus substantially earlier than the reported dates of 2007 (Nógrády et al., 2012) or 2008 (Aviv et al., 2014) for the emergence of MDR *S.* Infantis strain harbouring a large plasmid, even taking into account the 95% HDP of 1978 to 1995. It is, therefore, conceivable that after the initial acquisition of the pESI or pESI-like megaplasmid, such strains were circulating in the primary host reservoir at low prevalence before the proliferation of isolates that had the megaplasmid, perhaps due to changes in the host environment after which possessing the pESI or pESI-like megaplasmid bestowed a selective advantage upon such strains. In addition, as substantial levels of recombination have been detected to be occurring within the pESI or pESI-like megaplasmid (Tyson et al., 2021) and gene content of the pESI or pESI-like megaplasmid carried by different isolates has been shown to be variable (McMillian et al., 2020; Alba et al., 2023), there is a high possibility that genomic rearrangements, including the acquisition of novel genes, played an important role in the successful dissemination of this plasmid. BEAST phylogeny showed that the most recent common ancestor of the pESI or pESI-like megaplasmid-carrying isolates and isolates that did not have the megaplasmid, namely, a clade of 12 hierBAPS cluster 4 isolates from Brazil, E/W, Germany, and the Netherlands with a crown age of ~1986, was dated to ~1985. Hence, this result appears to indicate that the pESI or pESI-like megaplasmid-carrying isolates had diverged directly from this group of pESI or pESI-like megaplasmid lacking hierBAPS cluster 4 isolates and that the hierBAPS cluster 4 isolates may be the progenitors of the European and American isolates that harboured the pESI or pESI-like megaplasmid. Very interestingly, we also observed an evolutionary event, which is likely to have been initiated around 1999, that appeared to correspond to the loss of the pESI or pESI-like megaplasmid in eight German isolates (five from humans, two from cattle, and one from a wild animal) and in one Dutch isolate (part of the Europe-wide ring trials). These nine isolates were assigned to an ML tree clade (global phylogeny) that exclusively comprised isolates that did not have the pESI or pESI-like megaplasmid but also to hierBAPS cluster 4, which grouped 99 isolates with the pESI or pESI-like megaplasmid and 37 isolates without the megaplasmid. Therefore, hierBAPS clustering indicated that the isolates that the BEAST analysis suggested to have lost the pESI or pESI-like megaplasmid were of similar genetic background to isolates that carried the megaplasmid. Additionally, according to the cgMLST-based HierCC analysis, every one of these nine isolates belonged to HC20 cluster number 775, which in total comprised 29 isolates, none of which harboured the pESI or pESI-like megaplasmid. On the MSN, the nodes that comprised these nine HC20 cluster number 775 isolates were in close proximity to the nodes that represented the pESI or pESI-like megaplasmid-carrying isolates. Moreover, two isolates (both from Germany, one of which was indicated by the BEAST analysis to have potentially lost the pESI or pESI-like megaplasmid, whereas the other isolate was not part of the BEAST analysis) that belonged to HC0 cluster number 184347 and HC20 cluster number 775 formed the central node from which the branches leading to the nodes comprising the majority of the other HC20 cluster number 775 isolates and all isolates that were detected to carry the pESI or pESI-like megaplasmid extended from. The genetic relationship of the nine isolates that the BEAST analysis indicated to have lost the pESI or pESI-like megaplasmid to the megaplasmid-carrying isolates, as well as any potential mechanisms that had resulted in such plasmid loss, deserve further investigation since the presence of the megaplasmid is understood to provide an evolutionary advantage to the isolates that possess the pESI or pESI-like megaplasmid (Aviv et al., 2014; Alba et al., 2020; García-Soto et al., 2020). Augmenting the list of strains analysed with the time-measured phylogenetic methodology should be considered to improve the accuracy of the estimate of the locality and time point of the initial *S.* Infantis acquisition of the pESI or pESI-like megaplasmid. In particular, it will be vital to include *S.* Infantis isolates collected from the internationally dominant broiler and breeder lines as these appear to be the main vehicle responsible for worldwide dissemination of MDR *S.* Infantis, as well as *S.* Infantis isolates from countries where MDR *S.* Infantis (including pESI or pESI-like megaplasmid positive isolates) was initially reported such as Israel (Aviv et al., 2014), Japan (Yokoyama et al., 2015), Belgium, France (Cloeckaert et al., 2007), and Hungary (Szmolka et al., 2018), from both human and broiler sources. Consideration will need to be taken to review the availability of the WGS data corresponding to the isolate profile outlined above in public genomic data repositories; however, for *S.* Infantis, the amount and quality of such data have increased in recent years (Nagy et al., 2020).

According to our analyses, the isolates harbouring the pESI or pESI-like megaplasmid comprised the vast majority of the isolates in which one or more plasmids were identified, i.e., 194 of the 225 plasmid-carrying strains harboured pESI or pESI-like megaplasmid either by itself or with additional plasmid or plasmids. These isolates originated from several European localities, including the British Isles, Belgium, E/W, Germany, Hungary, Italy, Poland, and Scotland, as well as from the Middle East and from patients from the USA, including those patients with a travel history to Latin America. It is noticeable that the pESI or pESI-like megaplasmid-carrying isolates from patients from the USA and with travel history to Latin America as well as isolates from several European countries assigned to hierBAPS cluster 1, all but two of which had *bla*_CTX-M-65_, had a higher number of AMR genes compared to the pESI or pESI-like megaplasmid-carrying isolates from hierBAPS clusters 3 and 4. This output concurred with the results presented by Alba et al. (2020) and Tyson et al. (2021) for isolates harbouring the *bla*_CTX-M-65_ gene that carried the pESI or pESI-like megaplasmid. Generally, for isolates in our dataset that were previously analysed by other studies (Franco et al., 2015; Tate et al., 2017; Brown et al., 2018), there was an agreement regarding the presence of the pESI or pESI-like megaplasmid or other plasmids such as IncI1-I(Alpha) or pXuzhou21, although there were some differences in the nomenclature. In Franco et al. (2015), the pESI or pESI-like megaplasmid was termed pESI-like, and the IncI1-I(Alpha) plasmid was termed IncI1, whereas in Tate et al. (2017) the pESI or pESI-like megaplasmid was termed IncFIB and in Brown et al. (2018) this plasmid was termed IncFIB-like. Further support that our *in silico* plasmid detection was in agreement with the previous studies on some of the same isolates was that we did not find any Brazilian isolates harbouring the pESI or pESI-like megaplasmid. Indeed, three of our Brazilian isolates were previously analysed by Monte et al. (2019), where these isolates were also found not to possess the pESI or pESI-like megaplasmid. The presence of the IncR plasmid in two of these isolates that was reported by Monte et al. (2019) was confirmed by our study, although in the third isolate, we identified the presence of the Col(pHAD28) plasmid, which was not reported by Monte et al. (2019).

The *in silico* detected AMR genes and the resistance profiles that were detected in our study were also broadly congruent with the patterns of AMR gene presence as reported by previous studies, including studies that investigated some of the same isolates. In our study, the vast majority of the pESI or pESI-like megaplasmid-carrying isolates were found to harbour the *ant(3″)-Ia*, *sul1*, and *tet*(A) AMR genes and the *qacEdelta1* disinfectant resistance gene—a resistance gene pattern that was exclusive to isolates in which the pESI or pESI-like megaplasmid was detected and in agreement with the ESIr (Emergent *S.* Infantis resistance) AMR gene profile specified by García-Soto et al. (2020). Importantly, in the eight isolates sequenced with long-read sequencing technology that harboured the pESI or pESI-like megaplasmid, our analysis showed the localisation of the ESIr pattern genes to be on the pESI or pESI-like megaplasmid, thus confirming that the bacteria benefited from these important resistance genes by acquisition of the megaplasmid. Almost all of the isolates analysed in this study were ST32, and this was the case for both the isolates that carried pESI or pESI-like megaplasmid and those that did not. This pattern agreed with the results reported by García-Soto et al. (2020), where most of the ST32 isolates lacked the pESI or pESI-like megaplasmid but there were also a couple of ST32 isolates that were pESI or pESI-like positive. Moreover, as in the study by García-Soto et al. (2020), we found that all ST2283 isolates (*n* = 18) had the pESI or pESI-like megaplasmid and a single ST1032 isolate did not have this plasmid.

Brown et al. (2018) reported that American salmonellosis patients who were infected with *S.* Infantis harbouring the *bla*_CTX-M-65_ gene exhibited more severe symptoms and had more difficulties recovering from the disease. Here, the presence of *bla*_CTX-M-65_ was confirmed in all isolates that Brown et al. (2018) found carried this gene, all isolates except one reported by Tate et al. (2017), a single isolate reported by Franco et al. (2015), as well as two isolates from the British Isles, and one isolate from Scotland (all three from 2019). The presence of isolates with an AMR profile including resistance to third-generation cephalosporins in the UK is associated with imported products, or, in the case of human infections, can be associated with travel. An incursion is thought to have occurred in the case of three broiler farms in E/W (farms A, B, and C) with non-ESBL MDR *S.* Infantis harbouring the pESI or pESI-like megaplasmid that was linked by supplying a poultry processing plant (premises D) where similar isolates, originating from poultry meat products that included material sourced from elsewhere in Europe, had previously been identified. In addition, catching teams and cleansing and disinfection contractors operated on all three farms and hence were potentially implicated in the secondary dissemination of infection from the first farm. All three farms were subsequently confirmed as free of infection after intensive cleaning and disinfection inside and outside the poultry houses. In the case of farm A, this was achieved only after multiple crop cycles as the premises had been infected for a prolonged period before disinfection was intensified (Newton et al., 2020). As the MDR and ESBL-producing *S.* Infantis clone appears to be affecting the human population in E/W, potentially introduced via travel or from imported poultry products (Lee et al., 2021), this calls for constant vigilance and monitoring of the risk of transmission between the human and animal sources of this important pathogen.

Patterns of clustering of *S.* Infantis from five of the E/W premises of epidemiological interest, from which multiple isolates were sampled, indicated generally low levels of within-premise genetic diversity, including when comparing isolates sampled in different years, although there were some exceptions. All 16 isolates sampled from broiler farm A between 2013 and 2017 clustered in the same (E/W isolates only) ML tree clade on several shallow branches, indicating low within-premise and between-year genetic diversity at that farm. This conclusion was supported by the assignment of all 16 isolates to the same hierBAPS cluster, by the clustering of the SNP differences at the 10 SNP level where for ten isolates (2013–2107) the SNP address was 1.1.3.3.3.3.x, for five 2017 isolates it was 1.1.3.3.3.42.x, and for a single 2017 isolate it was 1.1.3.3.3.266.273, and by the cgMLST-based hierarchical clustering algorithm indicating that all farm A isolates were genetically similar with less than five-cgMLST allele difference (all isolates were assigned to the same HierCC group at the five-cgMLST allele HierCC threshold). There appeared to be even less genetic diversity amongst the four farm B isolates, which were assigned to the same ML clade as the 16 farm A isolates, had SNP addresses identical at the 5 SNP level (three isolates had SNP addresses identical at the 0 SNP level), and all were assigned to the same HierCC group at the two-cgMLST allele HierCC threshold (HC2 cluster number 1389). Therefore, the levels of within-premise genetic diversity amongst the isolates sampled from broiler farms A and B appear to be lower than what was described for *S.* Infantis isolates from several broiler farms in the Netherlands (Mughini-Gras et al., 2021) and are consistent with a single focal point of introduction. The laying farm E, on the other hand, showed evidence of the introduction of two genetically diverse but non-persistent *S.* Infantis strains onto those premises. A total of 36 farm E isolates, all sampled from layers in 2015 and 2016, displayed high levels of genetic similarity, clustering on a single shallow branch of the E/W only phylogenetic tree, sharing SNP address at the 0 or 5 SNP levels, and clustering within the same two- or five-cgMLST allele HierCC thresholds. Four 2015 isolates from farm E, none of which had the pESI or pESI-like megaplasmid, clustered in a different ML tree clade than the 36 farm E isolates described above, shared the SNP address with the 36 isolates only at the 250 SNP level and the HierCC cluster only at the 100-cgMLST allele threshold. The phylogenetic analysis, as well as the SNP address and HierCC clustering, indicated that the isolates sampled from processing plant D displayed the highest levels of within-premise genetic diversity of all the premises of epidemiological interest, which likely was due to the processing of birds from multiple sources, including imported chicken, at that facility.

Previous studies had linked the presence of the AMR genes to the accessory genome (i.e., plasmid derived) rather than being chromosomally encoded in the analysed isolates. This included the presence of many of the AMR genes that were also detected in our study on the pESI or pESI-like megaplasmid. In the present study, we were able to definitively identify the presence of an AMR gene on a plasmid in only very few cases (i.e., the AMR gene and the plasmid replicon type were present on the same contig of an assembled genome of an isolate) when the analysis was based on the short-read generated assemblies. Utilising the hybrid genome assemblies, ones that were based on both the short Illumina reads but also on the long Nanopore sequencing technology reads, revealed that the patterns of co-localisation of the AMR genes and plasmids on the same contigs were very much in agreement with previous studies. A hybrid assembly comprising both long- and short-sequence reads has been demonstrated to be very important for the accurate assembly of complex bacterial genomes (Becker et al., 2020), and in the present study, we have also shown that to unambiguously identify whether the detected plasmids harbour the AMR genes, long-read sequencing technology is an essential requirement.

In conclusion, multidrug-resistant *Salmonella enterica* subsp. *enterica* serovar Infantis isolates constitute a recently emerged and potentially highly severe public health threat. Such strains, often associated with the poultry production chain, have been reported from multiple countries worldwide, and many can be characterised as carriers of a large MDR plasmid termed pESI or pESI-like. Increasing our knowledge of the worldwide *S.* Infantis population structure can play an important role in understanding and preventing the further spread of this foodborne pathogen. Using the whole-genome sequencing data of 417 *S.* Infantis isolates originating from 15 countries/regions worldwide, but with a focus on England and Wales, the genomic clustering methods that we employed indicated that the analysed isolates grouped not by their country of origin or the host but rather by the presence/absence of the pESI or pESI-like megaplasmid. The reconstructed time-measured phylogeny provided novel insights into the initial acquisition of the pESI or pESI-like megaplasmid and its subsequent spread to different regions of the world. The patterns of the antimicrobial resistance gene presence harboured by the pESI or pESI-like megaplasmid-carrying isolates that were detected in our study were strongly in agreement with what has been reported in the literature; however, we have shown that to unambiguously identify whether the detected plasmids harbour AMR genes, long-read sequencing technology is an essential requirement.

## Data availability statement

The WGS data of the APHA-sequenced isolates presented in this study have been deposited on EnteroBase.

## Author contributions

RD, CT, LP, and JG conceived the study. JG performed population structure analyses and antimicrobial resistance gene and plasmid detection. JP performed the time-measured phylogenetic analysis. YT generated the SNP address data. RD, CT, and LP provided oversight and input into the interpretation of the data and results. JG, JP, RD, CT, and LP wrote the manuscript. All authors commented and approved the final manuscript, took public responsibility for appropriate portions of the content, and agreed to be accountable for all aspects of the study in terms of accuracy or integrity.

## Footnotes

^1^ https://github.com/tseemann/snippy

^2^ https://github.com/tseemann/snp-dists

^3^ https://github.com/tseemann/shovill

^4^ https://gt.rstudio.com/

^5^ https://card.mcmaster.ca/home
