# Supplementary material for: Geographical and temporal distribution of multidrug-resistant Salmonella Infantis in Europe and the Americas
Source: Front Microbiol. 2024 Feb 13;14:1244533. doi: 10.3389/fmicb.2023.1244533 (PMC10896835; doi:10.3389/fmicb.2023.1244533)
Supplement: Supplementary file 1 [file Data_Sheet_1.zip › Data Sheet 1/Supplementary Material.docx]

Supplementary Material

Geographical and temporal distribution of multidrug-resistant *Salmonella* Infantis in Europe and the Americas

Jaromir Guzinski^*^, Joshua Potter, Yue Tang, Rob Davies, Christopher Teale and Liljana Petrovska^*^

Animal and Plant Health Agency, Addlestone, Surrey, United Kingdom

*** Correspondence:**Jaromir Guzinski

[Jaromir.Guzinski@apha.gov.uk](mailto:Jaromir.Guzinski@apha.gov.uk)

Liljana Petrovska

Liljana.Petrovska@ukhsa.gov.uk

# Supplementary Data

## cgMLST and HierCC

The 417 *S*. Infantis isolates were grouped into 379 Sequence Types (STs) by EnteroBase based on their cgMLST allele profiles (Table S4). Thus, almost every isolate was assigned its own ST, underscoring low levels of clonality amongst the analysed isolates. The ST with the highest number of isolates was ST 184524 (eight isolates, all from Germany from human samples), followed by ST 52739 (seven isolates, all from E/W from animal feed), ST 176394 (six isolates, all from E/W from layers), and ST 184427 (five isolates, all from Germany from human samples). The remainder of the STs were assigned to three or fewer isolates, with 360 STs were assigned to a single isolate (Table S4).

The 379 STs were clustered into 75 clusters at the HC20 hierarchical level, six of which contained in excess of 20 isolates. The most numerous HC20 cluster was HC20 cluster number 7828 which was made up of 52 isolates, all of which carried the pESI or pESI-like megaplasmid, that were assigned to 46 unique STs (Figure S1, Table S4). The vast majority of these isolates were from E/W, with additional isolates from Germany and Italy. All of these isolates were assigned to hierBAPS cluster 4 and they clustered together on the ML tree as the orange clade (Figure 1). The second most numerous HC20 cluster was HC20 cluster number 343 that comprised 40 isolates (assigned to 39 unique STs) (Table S4). All of these isolates harboured the pESI or pESI-like megaplasmid (Figure S1). These were the USA and travel history from the USA isolates, two Scottish isolates, and two British Isles isolates that completely overlapped hierBAPS cluster 1 and grouped together as the light green clade on the ML tree (Figure 1). The third most numerous HC20 cluster was HC20 cluster number 422. This HC20 cluster comprised 32 isolates that were assigned to 25 STs, none of which were identified as pESI or pESI-like megaplasmid carrying isolates (Figure S1, Table S4). All of these isolates were assigned to hierBAPS cluster 4 and were grouping together on the ML tree as the blue clade, although a single isolate (Brazilian isolate SRR6927730 sampled from food) from this ML clade belonged to a different HC20 cluster (Figure 1, Figure S1, Table S4). Subsequently, HC20 cluster number 2398 was made up of 31 isolates that were assigned to 21 unique STs (Table S4). The majority of these isolates were from Germany, but also from the British Isles, E/W, the Netherlands, and from Brazil. None of these isolates carried the pESI or pESI-like megaplasmid and all of them belonged to hierBAPS cluster 5 and were part of the light violet ML tree clade (Figure 1, Figure S1, Table S4). HC20 cluster number 1369 comprised 29 isolates (assigned to 24 unique STs), all of which were pESI or pESI-like megaplasmid carrying isolates from E/W sampled from broilers or from animal feed (two isolates) (Figure S1, Table S4). HC20 cluster number 1369 isolates formed part of hierBAPS cluster 4 and completely overlapped with the pink clade of the ML tree (Figure 1). HC20 cluster number 775 was also made up of 29 isolates which belonged to 17 unique STs (Table S4). Almost all of these were from Germany, but also from the Netherlands (two isolates) and from Brazil (two isolates), and none of these isolates were detected to carry the pESI or pESI-like megaplasmid (Figure S1). Here again there was a high level of congruency between how the isolates clustered at the HC20 hierarchical level and the assignment of those isolates to hierBAPS clusters and their placement on the phylogenetic tree, as all 29 HC20 cluster number 775 isolates were assigned to hierBAPS cluster 4 and they occupied the neighbouring ML tree nodes such that 22 of these isolates completely overlapped with the cyan ML tree clade and the other seven isolates grouped amongst the 13 isolates which were not part of any distinct ML tree clade (Figure 1).

At the HC50 hierarchical level there were seven distinct clusters. HC50 cluster number 36, which grouped isolates both with and without the pESI or pESI-like megaplasmid, was by far the largest one with 305 isolates (Table S4). This HC50 cluster comprised all of the isolates from the 415 isolate ML tree with the exception of all light violet and all dark violet ML tree clade isolates (all hierBAPS cluster 5 isolates) and a single isolate from the red ML tree clade and hierBAPS cluster 3 (E/W isolate L00511-17 sampled from a dog which had the pESI or pESI-like megaplasmid) (Figure 1). Three HC50 clusters completely overlapped the light violet ML tree clade isolates and a single HC50 cluster grouped all of the dark violet ML tree clade isolates. Of the remaining two HC50 clusters, one comprised the two Dutch isolates (L01384-14 and S05257-14) that were identified as very highly genetically differentiated from all the other *S*. Infantis isolates, and the other HC50 cluster comprised solely the E/W isolate L00511-17. At the HC100 hierarchical level only two distinct clusters were formed, one with all of the 415 isolates presented on the ML tree on Figure 1 and other with the two highly genetically differentiated Dutch isolates L01384-14 and S05257-14 (Table S4).

The minimum spanning network (MSN) computed on the basis of the allele calls at the 3,002 cgMLST loci for 417 isolates was composed of 352 nodes, with each node representing one of HC0 clusters that the isolates were assigned to (Figure S1). Node size was proportional to the number of isolates making up each HC0 cluster. The topology of the MSN was broadly congruent with the topology of the 415 isolate ML tree (Figure 1), particularly in regards to the relative placement of isolates from the different localities of origin and isolates with and without the pESI or pESI-like megaplasmid. The upper portion of the MSN was occupied almost exclusively by isolates that had the pESI or pESI-like megaplasmid. Isolates that clustered in this section of the MSN were the isolates from the USA and isolates from the USA patients with travel history to Peru and Ecuador (HC20 cluster number 343) that formed hierBAPS cluster 1 and were clustering in the light green ML tree clade; a neighbouring MSN branch was occupied by isolates from hierBAPS cluster 4: E/W isolates from broilers and animal feed that belonged to the pink ML tree clade (HC20 cluster number 1369), isolates from Germany and the Netherlands (HC20 cluster number 7377), the Middle East (HC20 cluster number 229374 and 229375), and E/W (HC20 cluster number 1694) that all belonged to the black ML tree clade; followed by a branch (extending from a node belonging to HC20 cluster number 1373) that grouped isolates from Belgium, E/W, Germany, Hungary, Italy, Poland that all belonged to hierBAPS cluster 3 and the red ML tree clade; the subsequent MSN branch comprised hierBAPS cluster 4 isolates which harboured the pESI or pESI-like megaplasmid such as the Dutch, E/W, German, and Italian isolates from HC20 cluster number 7828 and 65093 that formed the orange ML tree clade

Closest to the nodes that represented the pESI or or pESI-like megaplasmid carrying isolates were the nodes representing several isolates that lacked the pESI or pESI-like megaplasmid that belonged to several different clades of the ML tree, such as German isolates (HC20 cluster number 775 and 85346), Dutch isolates from HC20 cluster number 775 and 180277, a Brazilian isolate from HC20 cluster number 114260 and an Italian isolate from HC20 cluster number 41803 (Figure S1). All MSN branches further down featured exclusively isolates that did not have the pESI or pESI-like megaplasmid. This included a branch occupied mostly by E/W and German isolates from HC20 cluster number 1695 that clustered on the ML tree as the green clade and that all belonged to hierBAPS cluster 2; a branch occupied by isolates from several different ML tree clades and different hierBAPS clusters that comprised mostly the isolates from E/W and Poland from the blue ML tree clade (HC20 cluster number 422), E/W isolates from the dark violet ML tree clade (HC20 cluster number 4421 and 178353), isolates from the British Isles, E/W, and an unknown locality (HC20 cluster number 43), and from the British Isles and E/W (HC20 cluster number 1292) from the green ML tree clade, and isolates from several localities from the cyan ML tree clade (HC20 cluster number 775); a branch featuring isolates from the light violet ML tree clade (hierBAPS cluster 5) that grouped isolates from Brazil, the British Isles, E/W, Germany, and the Netherlands (HC20 cluster number 2398), isolates from Germany (HC20 cluster number 99404), and Brazilian isolates from several different HC20 clusters; a directly opposite branch with isolates from the light violet ML tree clade: from HC20 cluster number 2398 (isolates from Brazil, E/W, and Germany), from HC20 cluster number 107369 (isolates from Brazil), and from HC20 cluster number 228981 (isolates from the British Isles, E/W, and from an unknown locality); and the final branch of the MSN that comprised light violet ML tree clade isolates from E/W from HC20 cluster 63284 (and a single isolate from HC20 cluster number 107401) as well as Brazilian isolates from HC20 cluster number 1987 and from several other HC20 clusters.

# Supplementary Figures and Tables

**Supplementary Figure 1** Minimum spanning network computed for the large geographical scale panel of 417 *S*. isolates Infantis based on their core genome MLST allele calls. The network comprises 352 nodes, one node for each of 352 HC0 clusters (hierarchical clustering of cgMLST level 0) the isolates were assigned to based on their cgMLST allele profiles. The node size is proportional to the number of isolates assigned to the same HC0 cluster. The nodes are coloured by the country of origin of the analysed isolates. The numbers inside the nodes correspond to the cluster identification numbers obtained after clustering the STs at the HC20 hierarchical level. Nodes with red outline comprised isolates that were detected to harbour the pESI or pESI-like megaplasmid. The length of all branches is scaled logarithmically.

**Supplementary Figure 2** Time-measured maximum clade credibility (MCC) tree from BEAST for 245 S. Infantis isolates. Years of the occurrence of key evolutionary events are specified on the tree and 95% height posterior density (HPD) is represented by purple bars for all phylogenetic splits. The tree is annotated by country of origin of the analysed isolates and the presence (yellow circle) or absence (white circle) of the IncFIB(pN55391) replicon type.

**Supplementary Table 1** 245 *S*. Infantis isolates selected for a time-measured phylogenetic analysis in BEAST.

**Supplementary Table 2** SNP distance matrix for the 415 isolates from the large geographical scale panel of *S*. Infantis isolates.

**Supplementary Table 3** SNP address for each of the analysed *S*. Infantis isolates (note, for some isolates it was not possible to obtain the SNP address).

**Supplementary Table 4** The assignment of the 417 isolates to different Sequence Types based on their cgMLST allele profiles, and clustering of the Sequence Types at different hierarchical levels: HC0 to HC2850.

**Supplementary Table 5** The *in silico* detection of the AMR genes, point mutations, and plasmids (or plasmid replicon types) for the 417 isolates based on the short-read assemblies. The co-localization of the AMR genes/point mutations and plasmids on the same contig, thus indicating that the AMR gene/point mutation was harboured by that plasmid, is specified by the contig labels that are in bold and the same colour of the text for the AMR genes and point mutation contigs (columns B to AW) and the plasmid contigs (columns AX to BM). The topmost row gives names of antimicrobial classes and the row below of antimicrobial compounds that each of the detected AMR genes conferred resistance to.

**Supplementary Table 6 A to E** IntegronFinder version 2 outputs for A) Complete class I integrons searched for in 417 Illumina sequenced isolates and detected in 196 isolates. B) CALIN class I integrons searched for in 417 Illumina sequenced isolates and detected in 21 isolates. C) Complete class II integrons searched for in 417 Illumina sequenced isolates and detected in eight isolates. D) Complete class I integrons searched for in 10 isolates additionally sequenced on the Nanopore and detected in nine isolates. E) Complete class II integrons searched for in 10 isolates additionally sequenced on the Nanopore and detected in six isolates. Integron gene content is detailed by specifying the contig number where each gene was found to be present on for all isolates that harboured integron(s).

**Supplementary Table 7 A and B** A) The total number of contigs, the number of fully circularized contigs, and the length (number of bases) of the four longest contigs for the hybrid assemblies utilizing both the long and the short reads for the ten *S*. Infantis isolates that were additionally sequenced with the Nanopore long read sequencing technology. Colour coding indicates which contigs were suspected to represent the core isolate genome, and which contigs comprised the pESI or pESI-like megaplasmid. B) The *in silico* detection of the AMR genes, point mutations, and plasmids (or plasmid replicon types) for the ten isolates based on the hybrid (long and short-read) assemblies. The co-localization of the AMR genes/point mutations and plasmids on the same contig, thus indicating that the AMR gene/point mutation was harboured by that plasmid, is specified by the contig labels that are in bold and the same colour of the text for the AMR genes and point mutation contigs (columns B to X) and the plasmid contigs (columns Y to AE). The topmost row gives names of antimicrobial classes and the row below of antimicrobial compounds that each of the detected AMR genes conferred resistance to.
