# Supplementary figures and images for: Geographical and temporal distribution of multidrug-resistant Salmonella Infantis in Europe and the Americas
Source: Front Microbiol. 2024 Feb 13;14:1244533. doi: 10.3389/fmicb.2023.1244533 (PMC10896835; doi:10.3389/fmicb.2023.1244533)

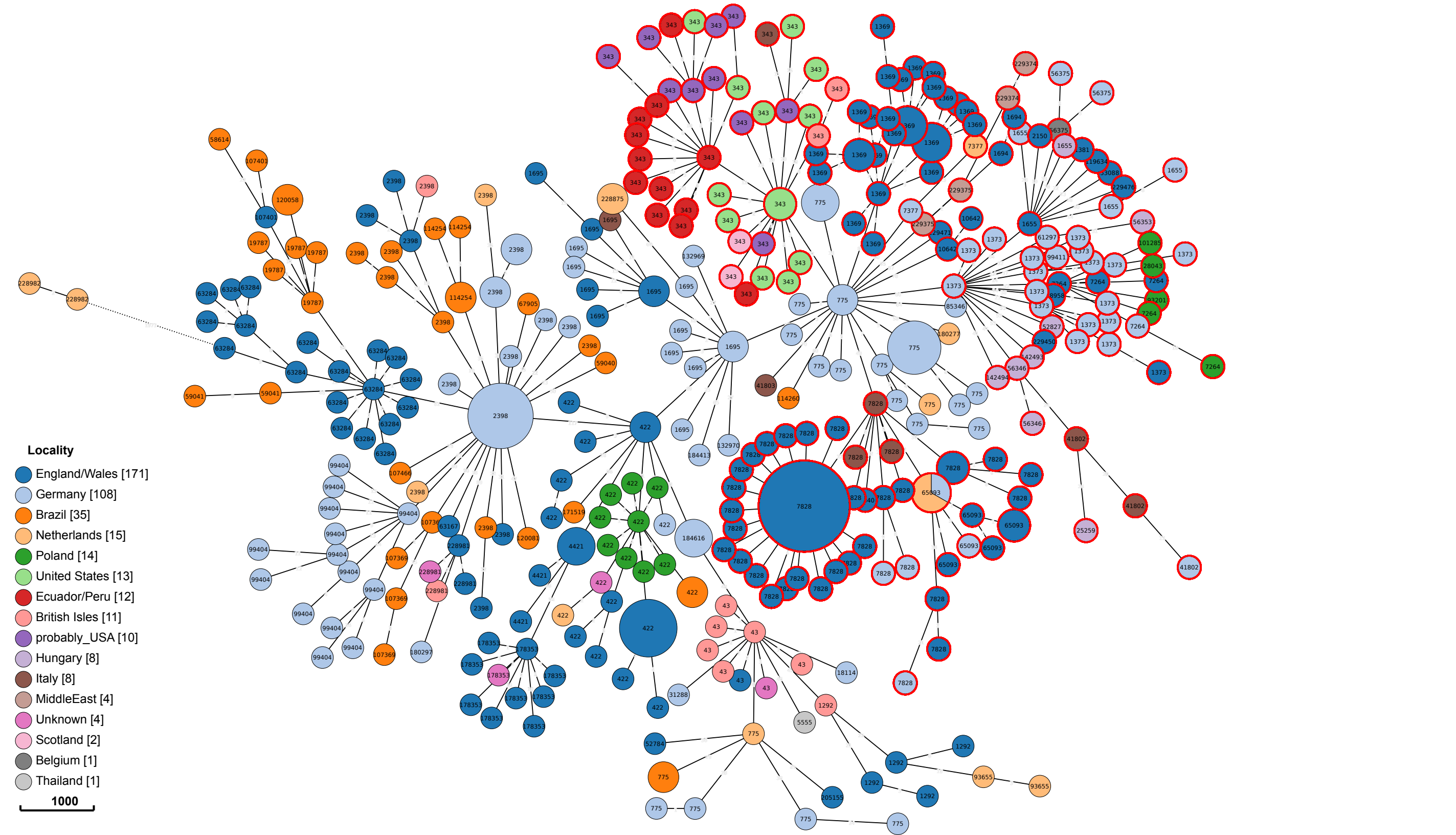

Supplement: Supplementary file 1 [file Data_Sheet_1.zip › Data Sheet 1/Supplementary Figure 1.PDF]

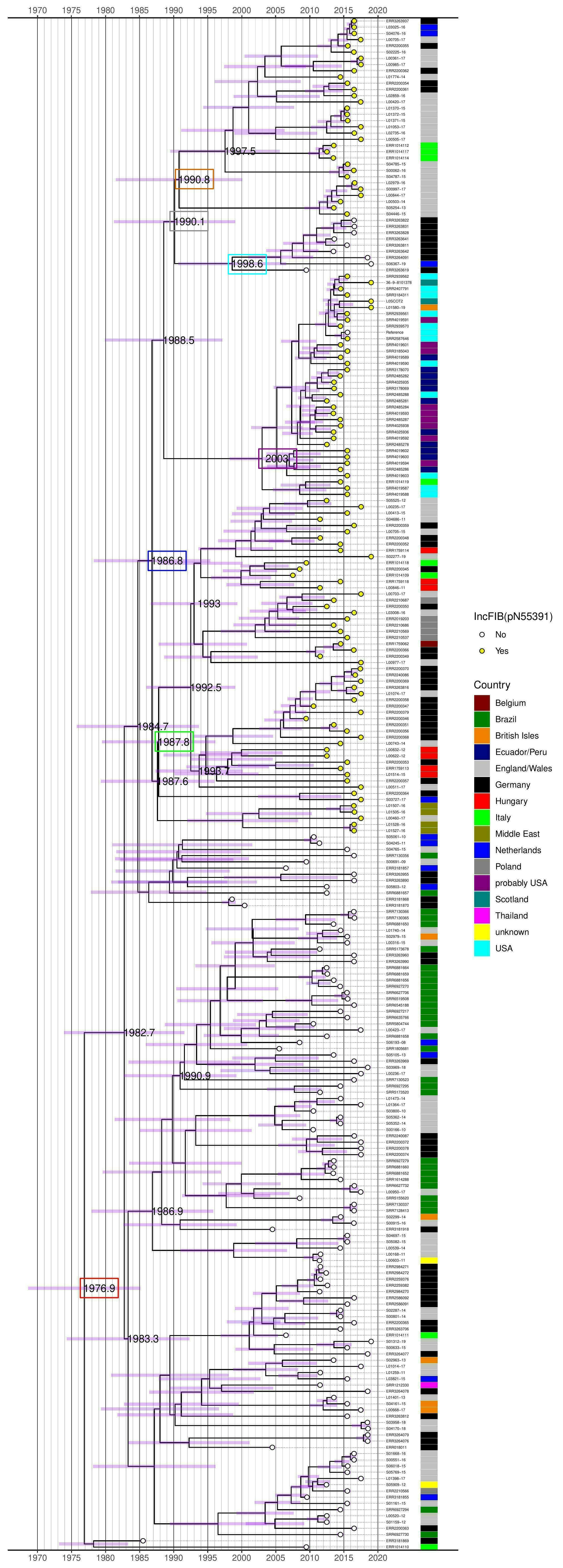

Supplement: Supplementary file 1 [file Data_Sheet_1.zip › Data Sheet 1/Supplementary Figure 2.JPEG]
